# Transforming modeling in neurorehabilitation: clinical insights for personalized rehabilitation

**DOI:** 10.1186/s12984-024-01309-w

**Published:** 2024-02-04

**Authors:** David J. Lin, Deborah Backus, Stuti Chakraborty, Sook-Lei Liew, Francisco J. Valero-Cuevas, Carolynn Patten, R James Cotton

**Affiliations:** 1 Department of Neurology, Division of Neurocritical Care and Stroke Service, Center for Neurotechnology and Neurorecovery, Massachusetts General Hospital, Harvard Medical School, Boston, MA USA; 2 Department of Veterans Affairs, Rehabilitation Research and Development Service, Center for Neurorestoration and Neurotechnology, Providence, RI USA; 3 Crawford Research Institute, Shepherd Center, Atlanta, GA USA; 4 Chan Division of Occupational Science and Occupational Therapy, University of Southern California, Los Angeles, CA USA; 5 Division of Biokinesiology and Physical Therapy, University of Southern California, Los Angeles, CA USA; 6 Department of Physical Medicine and Rehabilitation, UC Davis School of Medicine, Sacramento, CA USA; 7 Department of Veterans Affairs, Northern California Health Care System, Martinez, CA USA; 8 Department of Physical Medicine and Rehabilitation, Northwestern University, Chicago, IL USA

**Keywords:** Neurorehabilitation, Computational modeling, Neurorecovery, Rehabilitation engineering, Clinical translation

## Abstract

Practicing clinicians in neurorehabilitation continue to lack a systematic evidence base to personalize rehabilitation therapies to individual patients and thereby maximize outcomes. Computational modeling— collecting, analyzing, and modeling neurorehabilitation data— holds great promise. A key question is how can computational modeling contribute to the evidence base for personalized rehabilitation? As representatives of the clinicians and clinician-scientists who attended the 2023 NSF DARE conference at USC, here we offer our perspectives and discussion on this topic. Our overarching thesis is that clinical insight should inform all steps of modeling, from construction to output, in neurorehabilitation and that this process requires close collaboration between researchers and the clinical community. We start with two clinical case examples focused on motor rehabilitation after stroke which provide context to the heterogeneity of neurologic injury, the complexity of post-acute neurologic care, the neuroscience of recovery, and the current state of outcome assessment in rehabilitation clinical care. Do we provide different therapies to these two different patients to maximize outcomes? Asking this question leads to a corollary: how do we build the evidence base to support the use of different therapies for individual patients? We discuss seven points critical to clinical translation of computational modeling research in neurorehabilitation— (i) clinical endpoints, (ii) hypothesis- versus data-driven models, (iii) biological processes, (iv) contextualizing outcome measures, (v) clinical collaboration for device translation, (vi) modeling in the real world and (vii) clinical touchpoints across all stages of research. We conclude with our views on key avenues for future investment (clinical-research collaboration, new educational pathways, interdisciplinary engagement) to enable maximal translational value of computational modeling research in neurorehabilitation.

**Supplementary Information:**

The online version contains supplementary material available at 10.1186/s12984-024-01309-w.

## Background

On March 3rd and 4th 2023, the NSF DARE Conference (Transformative Opportunities for Modeling in Neurorehabilitation), co-hosted by the University of Southern California (USC) and the University of Washington (and with additional support from the National Institutes of Health), took place at USC in Los Angeles, CA. The conference brought together leading engineers, clinicians, computational scientists, rehabilitation researchers, representatives from the National Institutes of Health and the National Science Foundation, and client advocates to discuss the state of the science and identify transformative opportunities for computational modeling to advance neurorehabilitation.

The workshop was divided into four main sections (a) Modeling adaptation and plasticity, (b) Modeling for personalization, (c) Modeling human-device interactions, and (d) Modeling in the wild. There was a mix of keynote, platform, and poster presentations in which speakers were asked to address the following questions: *(i) What are challenges in your work related to neurorehabilitation that computational modeling can address? (ii) How has computational modeling supported your work, or could enhance your future work and what data are needed to support your modeling efforts? (iii) What is a key opportunity and challenge you see for the future of computational modeling in neurorehabilitation?*

As clinicians and clinician-researchers who attended this conference, here we offer our perspectives on the application of computational modeling to advance neurorehabilitation. Our overarching view is that clinical insight should inform the construction of and outputs from computational models in neurorehabilitation, and that this process requires close (and repeated) collaboration between researchers and clinicians. We start with two clinical case scenarios focused on upper extremity motor rehabilitation after stroke (inspired by real cases seen in a neurology clinic at a large academic medical center with dedicated, multidisciplinary neurorehabilitation specialists). We then discuss how computational models may, in the future, inform how we clinically approach these two distinct cases to maximize functional outcomes. We present seven fundamental discussion points to consider for clinical translation of computational models— (i) clinical endpoints, (ii) hypothesis versus data-driven models, (iii) biological processes, (iv) contextualizing outcome measures, (v) clinical collaboration for device translation, (vi) modeling in the real world and (vii) clinical touchpoints across all stages of research. As there is a notable ‘language’ gap between clinical and computational fields, we also aim to provide discussion to bridge these gaps. We conclude by providing our view on promising future directions for this exciting field.

## Main text

### Case 1

**Fig. 1:**
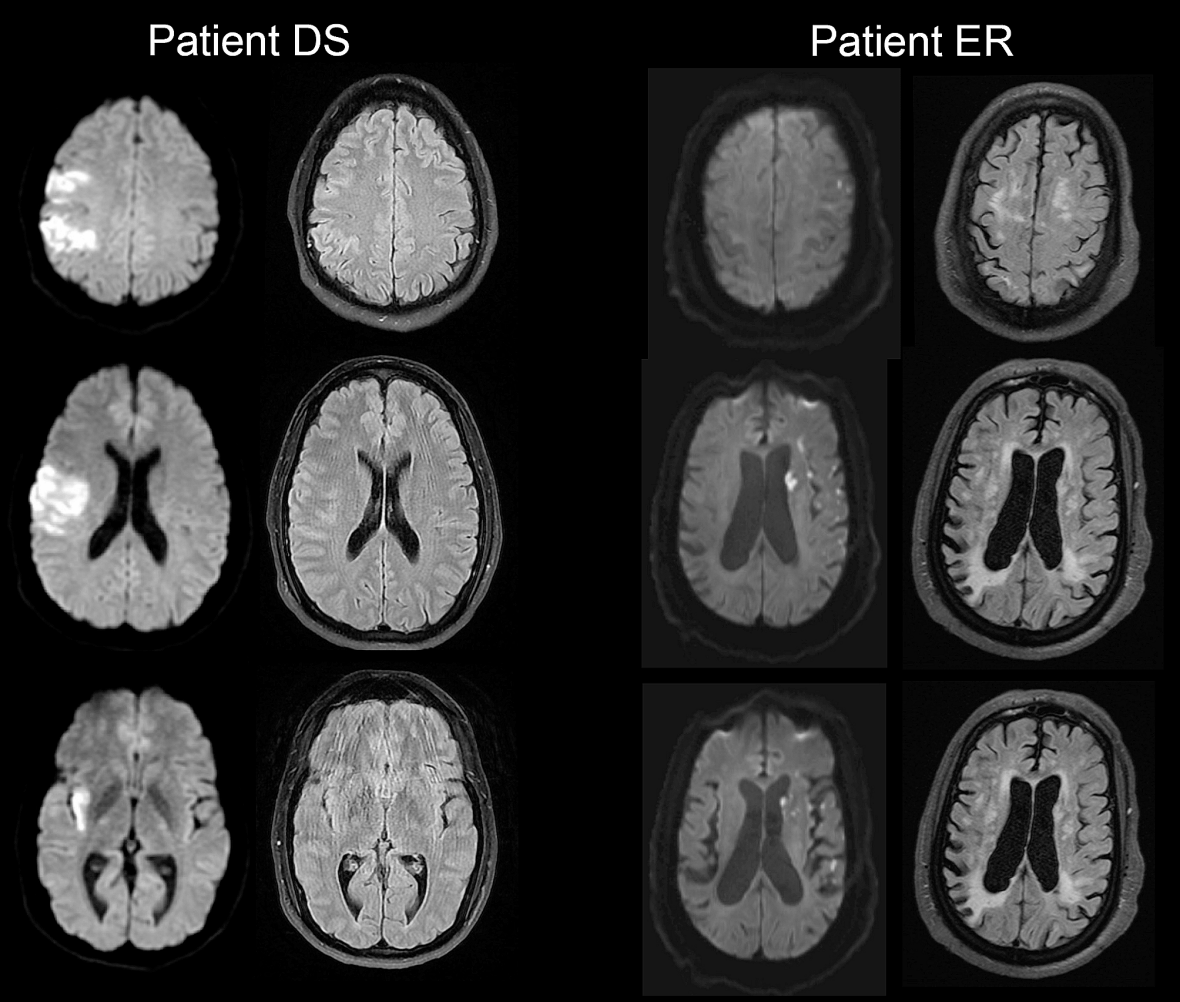
MRI Neuroimaging for Patient Case Presentations. Selected axial MRI diffusion weighted (left column) and fluid-attenuated inversion recovery (FLAIR) images for DS and ER patient case scenarios. These images were obtained at the time of acute stroke presentation (arrival of patients to the hospital). Bright areas on diffusion-weighted images indicate restricted diffusion (in these cases acute stroke). Note the R MCA distribution stroke for patient DS and the scattered L MCA distribution stroke for patient ER. Also note the severe leukoariosis (white matter hyperintensities in the periventricular areas) present for patient ER on FLAIR sequence

DS is a 38-year-old left-handed otherwise healthy man who presented with acute onset left-sided weakness, sensory loss, and difficulty speaking. He was found to have a stroke starting in the right sensory cortex (S2), extending downward through the corona radiata to the posterior insula, and involving the parietal and temporal operculum (Fig. 1).

His neurologic examination at the time of acute hospital discharge (day 4 post-stroke) was notable for dysarthric (slurred) speech, moderate expressive > receptive aphasia, complete left-sided sensory loss, left-sided neglect, and no left-sided motor strength. His upper extremity Fugl-Meyer (UE-FMA) score was 4 out of a total possible 66 points, representing severe hemiplegia (no movement) with intact reflexes. He was unable to ambulate. He was assigned a Modified Rankin Scale of five on discharge (severe disability, bedridden and requiring constant nursing care and attention). He was discharged to inpatient rehabilitation where he received 15 h per week of occupational, physical, and speech therapy for six weeks. He used an ankle-foot orthosis (AFO) and a quad cane to ambulate at home and required contact guard for these activities. He had substantial improvement in function while at inpatient rehabilitation and was discharged home with a plan to start outpatient therapy.

Three months after his stroke, his neurologic exam was notable for a mild expressive aphasia (difficulty speaking fluently), mild agrammatism, and moderate dysarthria. His upper extremity Fugl-Meyer motor component score had improved to 11 but still indicated severe hemiparesis. His sensory function remained impaired to light touch throughout the left hemibody and proprioception throughout the left hemibody. On gait assessment, while still requiring an AFO and quad cane, he was able to ambulate over short, level distances within the home without physical assistance but under family/caregiver supervision but without physical assistance.

At two years post-stroke, his upper extremity Fugl-Meyer had improved to 37, a 26 point change from his 3-month visit. He was using his left affected arm and hand mainly to support his unaffected side in doing activities of daily living. He continued to wear an AFO and was otherwise independent with gait (without the quad cane or other assistive devices) but continued to walk slowly and had difficulty in crowds. His language, while still slow, was functional at home, and he noticed continued improvements with speech therapy. He underwent implantation with Vagus Nerve Stimulation (a recently FDA-approved device for upper extremity rehabilitation [1]) and received 6-weeks of paired Vagus Nerve Stimulation-rehabilitation, after which his Fugl-Meyer improved by 5 points to 42 (out of 66 total points).

### Case 2

ER is a 68-year-old right-handed woman with extensive past medical history (diabetes, high blood pressure, high cholesterol, bilateral internal carotid artery atherosclerosis, prior stroke 10 years ago affecting her left-side from which she recovered). She was found by her husband unable to move her right side and unable to speak. MRI showed a shower of ischemic strokes in the left middle cerebral artery territory involving the left frontal and parietal lobes as well as the head of the left caudate and left insula (Fig. 1). There was additional evidence of a chronic right parietal infarct, multiple chronic lacunar infarcts, and severe white matter disease.

At the time of acute hospital discharge (ten days post-stroke), she was able to move her right upper extremity against gravity although she had limited coordination and clumsy finger and hand movements. She was able to name simple objects and repeat short phrases but was noted to have limited verbal output. She was transferred to inpatient rehabilitation, where she received three hours of OT, PT, and SLP (one hour each) per day for one month and was noted to have limited clinical improvement over this period. She was subsequently transferred to a skilled nursing facility for two additional weeks (documentation on frequency and duration of therapy during this time was unavailable) and subsequently discharged home. She received one month of home therapy (visiting OT, PT, SLP, two times per week), after which therapy was discontinued due to poor participation in activities and lack of clinical improvement.

Six months after her stroke, her examination was notable for very limited expressive and receptive language across all tasks presented. There were significant processing speed delays seen throughout the examination. Response to ideomotor and orobuccal praxis commands were delayed and atypical. In the right upper extremity, she had normal tone and full active range of motion with near full strength throughout on confrontational testing. She demonstrated perseveration and poor motor planning. In the lower extremities, she had full strength bilaterally. She had intact sensation to light touch and proprioception throughout. She required contact guard to close supervision assist. Recommendations were made to in her outpatient neurology clinic to attempt to re-initiate home therapy (OT, PT, SLP) with a focus on cognitive-motor interactions, but it was unclear how outpatient therapy or home services could be arranged given lack of access.

For further details of patient cases, see supplemental material 1.

### Summary of cases

These two cases, both focused on upper extremity motor impairments and recovery after stroke, are inspired by real-life cases seen in a multidisciplinary neurorehabilitation clinic. They illustrate important and generalizable points in clinical neurorehabilitation (for stroke but also for other acute neurologic injuries such traumatic brain injury, spinal cord injury) and, more broadly, about the realities of clinical care for patients with neurological injuries that are relevant to the context of computational modeling.

- There is a large degree of **heterogeneity** in patient presentation both acutely after stroke and in more chronic phases. In addition to initial presentation (i.e., severity of injury and size and location of stroke), there are a very large number of factors that influence recovery and response to rehabilitation such as pre-morbid functional status, co-morbid medical conditions, psychosocial factors, and acute interventions received. For instance, DS was a relatively healthy individual prior to his stroke with few risk factors, whereas ER was older, had several comorbidities, and several social determinants of health-related issues that might preclude her recovery to optimal levels of function. Taking these factors into account, what is their respective potential for recovery? Is it likely that they would respond similarly to the same interventions? What is needed to help them both respond optimally?
- There are **distinct time-based stages of recovery after neurologic injury**. Stroke recovery is typically divided into acute, subacute, and chronic phases, with each phase characterized by different biological processes [2]. Superimposed on different biologic phases of recovery is a **complex post-acute care continuum** [3] with changing clinical care teams and widely varying amounts and types of rehabilitation therapy administered [4]. Moreover, access to clinical rehabilitation is variable and influenced by a number of factors including geographic location, socioeconomic circumstances, insurance, personal factors, and support [5]. DS and ER both transitioned through different care settings (acute stroke hospitalization, inpatient rehabilitation, skilled nursing facility) in the acute and subacute phases of recovery and had different access to outpatient rehabilitation in the chronic phase. Would intervening sooner and with more intensity have had a positive impact or negative on each of these patients? What is the best combination of rehabilitation interventions that we could make available to DS and ER during each phase of recovery that will lead to the greatest improvements for each of them?
- **Different neurologic deficits** (motor, sensory, cognitive, language) recover to different degrees and with different time courses after neurologic injury, indicating that resolution of the neurovascular injury and plasticity differ across the brain’s neural systems (vis-à-vis different neuroanatomical locations) after stroke (**modality-specific recovery**) [6, 7]. There are interactions among deficits and their recovery, that reflect interactions amongst these neural systems. There are further interactions between neurologic variables, and premorbid health as well as social determinants of health [8, 9]. For instance, DS had a right middle cerebral artery stroke, presenting with language and speech motor deficits, left-sided weakness, sensory loss (he is a rare case of left-hand dominant with language centers localized to right hemisphere). Language, speech, and sensory function recovered well over time while motor function remained impaired despite intense rehabilitation. ER, in the context of multiple co-morbid medical conditions and prior stroke, had a left middle cerebral artery stroke, presenting with language and right-sided motor deficits. Her primary motor deficits recovered very well but her functional recovery was limited by cognitive and motor planning impairments. Capturing data related to these neurologic variables and baseline personal characteristics, in addition to the type, timing and dosing of interventions, would provide valuable insights to inform how to manage DS and ER most effectively for the greatest value and positive outcomes.
- **Outcome measures** in neurorehabilitation are not currently systematically gathered or standardized as part of clinical practice. Similarly, current approaches to rehabilitation therapy are **not quantified or standardized** [10]. Outcome measures in DS’s courses were captured and entered into a database as part of a research study; there was a lack of quantitative outcomes during ERs recovery course. There are limited current efforts to standardize and pool rehabilitation data. Would a standardized approach to clinical neurorehabilitation that included quantitative outcomes data collection create new insights and strategies to ultimately improve outcomes for DS and ER?

## Key questions and discussion

Should we treat the two presented patients (DS and ER) differently? This is a critical question for practicing rehabilitation clinicians. The therapy a given person receives is, by definition, personalized — clinicians are trained to consider individual factors and goals when working with their clients. However, we do not yet have a systematic and causal evidence base to consistently tailor rehabilitation treatments to specific patients to optimize outcomes. Such an evidence basis is required to move from unstructured personalization to a systematic and structured precision rehabilitation approach. This represents a key challenge and opportunity for computational modelling in neurorehabilitation. Altogether, our cases and discussion lead to a fourth and critical question for the application of computational modeling to neurorehabilitation: ***(iv) how can computational modeling help build the evidence base for precision neurorehabilitation*?** Specifically, what structure and level of details should computational modeling approaches have? Moreover, how should we collect, analyze, and model neurorehabilitation data so that, in the future, clinicians can be informed of the most likely therapies and therapeutic parameters (e.g., dose, frequency) that might be most likely to support optimal recovery for individuals such as DS and ER? Here we put forward **seven discussion points** (Table 1) for computational modeling projects that we think will be critical for this effort. The first four are suggestions for computational neurorehabilitation modelers to improve the translatability of their work for greater clinical relevance, and the last two are opportunities for increased collaboration.

**Table 1:** Key points for clinical translation of projects involving computational modeling in neurorehabilitation

• Identify your ultimate clinical endpoint, even if translation is far in the future
• Distinguish between hypothesis-driven and data-driven models
• Be precise about hypothesized biological processes and levels of abstraction
• Understand and contextualize outcome measures
• Clinical and computational collaboration are necessary to move neurorehabilitation devices into the clinic
• Modeling rehabilitation data “in the wild” will introduce new sources of variability but is essential for clinical translation
• Increasing clinical touchpoints (data collection, device testing, brainstorming and discussion) is a good research investment

### Identify your ultimate clinical endpoint, even if translation is far in the future

For modeling in neurorehabilitation, it is important to keep in mind the intended clinical endpoint. Is the ultimate goal a better way to diagnose or stratify patients (i.e., cluster groups of patients into those with similar characteristics for clinical trials)? Or is the goal to advance or develop a therapy (i.e., optimize parameters of brain stimulation [11])? Note, of course, that there may be very valuable fundamental modeling that is not immediately related to a clinical endpoint. Fundamental modeling, for example of intracortical neural dynamics [12] or of corticospinal motor control [13], could pave the way for more informed rehabilitation therapies in the future. We propose, whether the model has immediate or more distant clinical application, there is consequential value to explicitly stating the eventual clinical application (e.g., improving brain-computer interface decoding for assistive use in people with paralysis [14–16] or optimizing rehabilitation protocols [13]). Something as simple as starting a modeling project with the statement: “The ultimate clinical goal of this computational model is to …”, would be incredibly helpful for translating utility between computational modelers and clinical partners.

### Distinguish upfront between hypothesis-driven and data-driven models

Big data collection in medicine can be used as either a means to perform parameter estimation for a hypothesis-driven or as purely statistical (data-driven) representation of the condition or patient [17]. The hypothesis-driven approach generally contains equations that encode hypothetical causal mechanisms or interactions that need to be tuned (i.e., to estimate the true values of the parameters of the model) to a particular patient or situation. These models are, in fact, the computational representation of a predictive hypothesis. For example, one recent study simulated a neural network of corticospinal neurons controlling finger extension by pre-specifying relationships between corticospinal neurons, motor neurons, and finger extension torque [13]. The ultimate goal was to study mechanisms of cortical reorganization after stroke and better parameterize rehabilitation protocols. Data-driven models are more of a “black-box” approach where statistical or regression models, artificial neural networks, etc. use large amounts of data to represent a population in general, and the relationship of a particular patient to a general population of patients. As such, they are descriptive representations that can be of value, but lack a pre-defined mechanistic hypothesis. If the underlying mechanisms are known, hypothesis-driven models are preferred. Data-driven models can also be very useful, especially for providing a starting point to understand key relationships.

### Be precise about hypothesized biological processes and levels of abstraction

The DARE conference featured a number of important talks dedicated to adaptation, learning, and plasticity. Here we emphasize the importance of using explicit terminology, which will inform parameters of equations that model these processes. “Plasticity” has become a widely used and somewhat catch-all term referring to topics ranging from biological to phenomenological. Perhaps the most well-known type of plasticity is known as Hebbian, referring to the synaptic principle that “neurons that fire together, wire together” [18, 19]. Mechanisms of Hebbian synaptic plasticity have been shown to be regulated by millisecond resolution spike-timing dependent rules [20]. Indeed, some forms of brain stimulation for neurorehabilitation are thought to operate via such mechanisms [21]. And there is promise in recent treatment protocols for rehabilitation based on these principles [22]. But one should be reminded that not all plasticity underlying experience-dependent change is Hebbian [23, 24]. Similarly, learning encompasses a wide range of phenomena ranging from low-level mechanisms to high-level cognitive decisions [25]. Adaptation is one form of learning but there are other forms as well, each engaging distinct neural substrates [26]. Being precise and specific about the plastic and learning processes involved in a proposed rehabilitation treatment (and thus explicitly defining the variables and parameters for computational models to predict outcomes) will lead to greater interpretability of results, rationalization for why different patients (D.S. and E.R.) might respond differently to the same treatments, and help build the translational knowledge base underlying clinical rehabilitation.

### Understand and contextualize outcome measures

There is a large current emphasis in neurorehabilitation on outcomes data collection; big data frameworks will undoubtedly lead to very valuable insights for neurorehabilitation [27, 28]. When modeling data, key attention to specific outcomes and their context is extraordinarily important. For example, one recent study showed that the Modified Rankin Scale, a very commonly used outcome measure for acute stroke trials, does not distinguish differences among or clinically meaningful changes within rehabilitation-related outcomes [29]. One useful framework for contextualizing outcomes is the International Classification of Functioning, Disability, and Health (ICF) classification system [30] that structures functioning and disability in different hierarchical levels: (i) body structure/impairment, (ii) activity limitation, and (iii) participation restriction. For upper extremity hemiparesis after stroke, these levels would correspond to, respectively, (i) loss of strength and motor control, (ii) decreased ability to complete an action or task, and (iii) decreased involvement in work, productivity, or social situations leading to diminished quality of life. Note that levels of the ICF can uncouple from each other — that is, while impairment and activity and participation are all broadly associated, they can dissociate. In the case of participation, a patient with complete limb loss can continue to participate, be productive at work, and engage in social activities with assistive equipment to enjoy a good quality of life. The uncoupling of activity limitations and impairment are a bit more subtle but very relevant to modeling. The difference can also be stated as distinguishing between compensation (using abnormal movement patterns to accomplish a task) versus restitution (true neural and behavioral recovery). For example, to successfully accomplish the same reaching task, a patient could gain additional degrees of elbow extension (restitution, minimizing impairment) or flex the whole trunk forward to reach the distance (compensation) [31]. Developing computational models (i.e., using kinematic data) that can differentiate between restitution and compensation would be incredibly valuable [32]. Overall, when modeling data, it is critical to consider what the data being collected in rehabilitation represent and ideally establish models that span and link ICF levels [33]. 

### Clinical and computational collaboration are necessary to move neurorehabilitation devices into the clinic

Devices hold tremendous promise for neurorehabilitation given their flexible programmability, but clinicians and computational neuroscientists need to work together to understand their capabilities and limitations and to figure out how to personalize them. A quick read of one of the largest upper limb stroke rehabilitation robotic trials performed to date would seem to indicate that robots have limited efficacy for improving outcomes [34]. However, a closer read would reveal that robots effectively deliver high-repetition training at doses that are impractical to deliver in current clinical care: the robots in the VA-ROBOTICS trial delivered over 1000 movement repetitions per session, an order of magnitude above the $\sim$ 30 known to be achieved in current clinical care [35]. Other large scale clinical trials have shown similar overall results [36]. These recent large-scale trials have been successful in harmonizing protocols and outcome measures across sites (to achieve their relatively large N) [37]. But large, multi-site trials of devices (including robots) have not yet been able to achieve *personalization* to patient-specific characteristics (patient characteristics or patterns of brain injury) [38]. That is, D.S. and E.R. would likely require very different robotic training protocols to maximize their recovery. Nor have large N robotic trials advanced principles that are fundamental to plasticity and learning. Recent smaller sized trials [39–41], which featured delivering upper extremity robotic therapy to improve movement quality, have shown promise in these directions but require further validation. In addition, robots (and other devices) have the capability to rigorously assess patient-specific impairments and distinct aspects (dexterity, spasticity) of the stroke hemiparesis phenotype [42–47]. We predict such evidence-based personalization (systematic assessments of deficits coupled with delivery of specific movement patterns) will be required for robotic rehabilitation studies to show greater efficacy than standard of care. Importantly, multiple comparisons of conventional vs. robot-assisted therapies in stroke rehabilitation fail to show a clear advantage of one over the other. Additionally, the practical logistics of integrating robotic therapies to clinical practice are often under considered, which highlights the need for early optimization of this through close interactions between engineers and clinicians [48]. Overall, robots and other devices hold tremendous promise for neurorehabilitation given their programmability, but it will take future research (and close clinical collaboration) to determine how to best program them to optimize outcomes.

### Modeling rehabilitation data “in the wild” will introduce new sources of variability but is essential for clinical translation

One of the main DARE conference themes was dedicated to efforts of “modeling in the wild”. Topics ranged from in-clinic, video-based analysis of gait to large-scale data collection using wearable sensors. As clinicians working in neurorehabilitation, we agree with the value of gathering data from real world settings, including medical clinics, therapy sites, and from home and in the community. These efforts will really be “in the wild” because, as those engaged in such efforts will quickly realize, experiments based in clinical settings are always less controlled with more unforeseen challenges and sources of variability. We hope that researchers will come to embrace these challenges as opportunities because even though they may seem logistically overwhelming (i.e., a rehabilitation clinic without an IRB committee to approve research), these issues are at the very core of clinical translation of neurorehabilitation research; addressing them will move the field forward. While there is a plethora of outcome measures designed to measure different constructs within the ICF, it is ultimately real-world data that will either endorse their construct validity. We may find some laboratory measures have limited real world meaning. In addition to data collection in clinical settings, we also think there is high value to testing devices in the clinic, even in their prototype stage, as it can frequently reveal practical issues that become the most important challenges or help identify the most impactful questions [49]. Finding the time and venue to do this type of testing and iteration of devices is of high importance and great translational value. Establishing rigorous data collection around the process of clinical device testing in a pragmatic way can also accelerate the growth of the evidence-base. Collecting data in naturalistic settings will inevitably pose new challenges to computational models, mainly in accounting for the (innumerable) sources of real-world variability. For example, one might imagine that device-testing with D.S. (with a more pure motor and language stroke presentation) would be very different that for E.R. (in whom social determinants of health and cognitive factors would be significant considerations). In our view, this represents an opportunity because, once the framework for data collection and modeling has been established and sources of variability have been defined, data collection via sensors and other ecological monitoring devices can generate rich datasets and provide new insights [50]. 

### Increasing clinical touchpoints (data collection, device testing, brainstorming and discussion) is a good research investment

For computational modeling to have an impact on clinical neurorehabilitation, there should be more regular interaction between researchers engaged in computational modelling research and practicing neurorehabilitation clinicians. Optimal translation of new knowledge to the clinic applies best practices for knowledge-to-action translation, which can only be achieved with active involvement of clinical perspectives from start to finish of research projects [51–53]. Interactions start in research and clinical training programs. Clinical versus research training are often presented as mutually exclusive pathways and careers. Clinician-scientist training pathways are prolonged and have difficulty retaining people after training [54]. In addition, clinician-scientists most often end up dedicating most of their time to research (i.e., 80%) as this is what is required to be competitive for research grant funding [55]. Clinical training (i.e., MD, PT, OT, SLP) could incorporate more curricula dedicated to research data collection, analysis, and modeling. Research training in neurorehabilitation (i.e., Masters, PhD, or Post-Doc) could involve more clinical touchpoints, which might take the form of dedicated time spent in clinical settings (i.e., observing clinic sessions or inpatient rounds).

We propose establishing dedicated time in proposal requests and budgets for clinicians to be engaged and provide perspectives on research projects (e.g., 20% as a clinical consultant). In addition, dedicated (and funded) time for researchers to engage clinically— discussing outcomes measures, testing devices, discussing clinical goals— would be a worthwhile investment. Time spent by researchers observing and engaging with patients (e.g., researchers directly observing D.S. and E.R. while engaged in rehabilitation therapy) would be meaningful and helpful for continued clinical grounding. We also propose more funding opportunities specifically promoting these connections, collaborations, and early translational interactions; these could take the form of program projects that require effort from both computational modelers and clinicians. In addition to including these opportunities in requests for applications, these opportunities could be integrated into individual (NIH K award, VA Career Development Award, etc.) and institutional (NIH T32 and NSF Research Traineeship) research training programs. Furthermore, more (and regular) conferences like the NSF DARE conference, engaging both computational modeling researchers and clinicians, are highly valuable. These venues will further the prolonged interdisciplinary interactions between researchers and clinicians that are critical to promoting shared language and goals. Such alignment will translate to computational modeling resulting in better diagnosis and treatment and ultimately, better patient outcomes.

## Conclusions and future directions

Computational modeling holds great promise for the field of neurorehabilitation. Clinical experience supports that there is a large degree of heterogeneity in patient presentation and recovery, that there are distinct time windows of recovery upon which a complex rehabilitation care continuum is placed, that deficits recover in a modality-specific manner, and that current outcome measures and approaches to neurorehabilitation therapy are not routinely gathered or standardized. With that said, rehabilitation therapy is already, by definition, personalized— rehabilitation specialists are trained to account for individual factors of their patients when planning the rehabilitation program (e.g., planning to discharge someone in a power wheelchair to inaccessible housing is unlikely to produce a good outcome). However, we continue to lack a systematic evidence-base to inform what therapies or parameters of therapy might be best integrated into personalized rehabilitation efforts. In the process of building this evidence base, we may find the different comorbidities, stroke etiologies and regions, social determinants of health, or other neurophysiologic or behavioral biomarkers tell us that our two example patient cases (DS and ER) should receive distinctly different rehabilitation plans including pharmacological, therapeutic and device prescriptions to maximize their individual long-term outcomes.

A computational modeling framework could thus help build the evidence base for personalized therapy to improve outcomes for patients: how can we collect, analyze, and model neurorehabilitation data to deliver specific therapies to specific patients to improve outcomes? A computational model is, at its core, a hypothesis, which can be used to make experimental predictions, such as how a patient might respond to a form of rehabilitation [13]. Creating these models for neurorehabilitation is particularly challenging, given the need to model our interventions’ influence across *time, throughout the nervous system*, and across *levels of the ICF.* How does a single repetition of a therapy in the subacute period promote short term plasticity, where does it occur, how does it change impairment now, how does that alter the dynamically updated therapy plan, and how does this alter upper extremity use in the real world a year from now? Computational models that account for these complexities, including dynamics over time [56], are critical to progress in rehabilitation. As clinicians, we propose that the following perspective points are important considerations for computational modeling research: (i) clinical endpoints, (ii) hypothesis- vs. data-driven models, (iii) biological processes, (iv) contextualization of outcome measures, (v) clinical collaboration, (vi) modeling in the real world, and (vii) clinical touchpoints across all stages of research.

Overall, we assert that all stages of computational modeling efforts (from data collection to device testing to model refinement) would benefit from clinical touchpoints— engagement of clinicians and researchers together in the process. Patients and their caregivers should also be engaged in the rehabilitation research process from early stages; their perspectives will add enormous value [57]. Furthermore, if we are truly to achieve better patient outcomes, collaborations should be across clinical settings globally and not just in developed or high-income countries. The key for the field will be identifying incentives for all parties to continue to engage, especially given the demands of clinical work and academic research. This will likely involve aligning incentive structures at multiple stages from national policy and funding decisions, clinical spaces designed to facilitate translational research, administration that understands and supports the work, and both local and global communities encouraging prolonged, cross-disciplinary interactions.

### Electronic supplementary material

Below is the link to the electronic supplementary material.

Supplementary Material 1

## Data Availability

More information on the 2023 NSF DARE conference can be found at https://sites.usc.edu/dare2023/events/.

## Abbreviations

NSF: National Science Foundation
DARE: Disability and Rehabilitation Engineering
USC: University of Southern California
tPA: Tissue plasminogen activator
MCA: Middle cerebral artery
MRI: Magnetic resonance imaging
UE-FMA: Upper extremity Fugl-Meyer
MRC: Medical research council
AFO: Ankle foot orthosis
OT: Occupational therapy
PT: Physical therapy
SLP: Speech and language pathology
ICF: International Classification of Functioning
