# Supplementary material for: Transforming modeling in neurorehabilitation: clinical insights for personalized rehabilitation
Source: J Neuroeng Rehabil. 2024 Feb 4;21:18. doi: 10.1186/s12984-024-01309-w (PMC10840185; doi:10.1186/s12984-024-01309-w)
Supplement: Supplementary file 1 — Supplementary Material 1 [file 12984_2024_1309_MOESM1_ESM.docx]

SUPPLEMENTAL MATERIAL

Case 1

DS is a 38-year-old left-handed man with a history of attention deficit hyperactivity disorder, who presented with acute onset left-sided weakness, sensory loss, and difficulty speaking. He did not meet eligibility for acute stroke therapy (did not arrive at the hospital within the time window (up to 4.5 hours) for tissue plasminogen activator (tPA) and the carotid artery was fully occluded preventing endovascular therapy). He was found to have a right internal carotid artery dissection and a right superior division middle-cerebral artery (MCA) territory ischemic stroke (stroke mechanism was artery-artery embolism). Magnetic Resonance Imaging (MRI) showed that the stroke started in the right sensory cortex (S2), extended downward through the corona radiata to the posterior insula and involved the parietal and temporal operculum (Figure 1). He was briefly admitted to the intensive care unit for blood pressure management. No clear inciting factors were found to explain the carotid dissection.

His neurologic examination at the time of acute hospital discharge (day 4 post-stroke) was notable for dysarthric (slurred) speech, moderate expressive > receptive aphasia, complete left-sided sensory loss, left-sided neglect, and no left-sided motor strength. He was enrolled in a natural-history research study of motor recovery (clinicaltrials.gov NCT03485040, which required research consent) and, in this context, an upper extremity Fugl-Meyer (UE-FMA, motor component) was performed and documented by trained research staff. His UE-FMA score was 4 out of a total possible 66 points, representing severe hemiplegia (no movement) with intact reflexes. He was assigned a Modified Rankin Scale of five on discharge (severe disability, bedridden and requiring constant nursing care and attention). He was discharged to inpatient rehabilitation where he received 15 hours per week of occupational, physical, and speech therapy. He remained at this facility for six weeks. He had substantial improvement in function during this time. Per notes obtained from the inpatient rehabilitation facility at the time of discharge home, he had 3/5 strength (MRC grade) on shoulder abduction, 2/5 strength on bicep flexion, and 1/5 strength on finger flexion (all improved from 0/5 on rehabilitation facility admission, representing no movement at that time). He used an ankle-foot orthosis (AFO) and a quad cane to ambulate at home and required contact guard for these activities. In the community, the discharge recommendation was to use a wheelchair for mobility. He was discharged home with a plan to start outpatient therapy. His 65-year-old mother came to live with him, together with his wife and young children, to provide additional support.

DS was seen three months after his stroke in a multidisciplinary neurorehabilitation clinic. The week prior he had started outpatient occupational, physical, and speech therapy (OT, PT, SLP) near his home. He still required help for dressing, toileting, and showering. He stated that one of his main goals was to be able to help his family with cooking; he felt like a burden on his family members. His neurologic examination was notable for being awake, alert, pleasant, and cooperative. He had a mild expressive aphasia (difficulty speaking fluently), mild agrammatism, as well as moderate dysarthria. In the left upper extremity, he had increased tone (i.e., resistance to passive movement as measured by the Modified Ashworth Scale scoring 2+), most prominent at the fingers. He could lift his left upper extremity above his head in a flexor-synergy pattern. It took significant effort to extend the elbow and he could only achieve about 10 degrees of active extension from his baseline flexed position. He could not pronate or supinate the forearm. He had 20 degrees of wrist flexion and was just beginning to develop trace extension beyond neutral. He could not extend his fingers independently. His upper extremity Fugl-Meyer motor component score (obtained again in context of the research study) had improved to 11 but still indicated severe hemiparesis. His sensory function remained impaired to light touch throughout the left hemibody and proprioception throughout the left hemibody. On gait assessment, while still requiring an AFO and quad cane, he was able to ambulate over short, level distances within the home without physical assistance but under family/caregiver supervision but without physical assistance. He demonstrated left foot circumduction through swing, limited knee flexion and flat foot landing as well as limited push off. Recommendations were made to start a medication to treat spasticity (tizanidine) and work with therapists on incorporating his affected upper extremity into daily activities, particularly those that were most important to him (like helping with cooking).

The interdisciplinary clinic continued to follow DS clinically over the course of three years with visits approximately once every 6 months. He continued with intensive therapy (3-4 times per week, OT, PT, SLP) throughout this time, often self-paid as his insurance only covered a few visits per year. At two years post-stroke, his upper extremity Fugl-Meyer was documented at 37 (an improvement of 26 points from his 3-month visit). He had moderate spasticity (Modified Ashworth Scale 2+). He was using his left affected arm and hand mainly to support his unaffected side in doing activities of daily living. He continued to wear an AFO and was otherwise independent with gait (without the quad cane or other assistive devices) but continued to walk slowly and had difficulty in crowds. His language, while still slow, was functional at home, and he noticed continued improvements with speech therapy. He underwent implantation with Vagus Nerve Stimulation (a recently FDA-approved device for upper extremity rehabilitation [3]) and received 6-weeks of paired Vagus Nerve Stimulation-rehabilitation, after which his Fugl-Meyer improved by 5 points to 42 (out of 66 total points).

Currently, DS lives with his wife and two children (his mother moved away) and is contemplating return to work as a software engineer. He is working with his company and providers to determine what accomodations could be provided given that his continued loss of function on his dominant side as well as his communication disorder. He continues to be on the lookout for any new and available therapeutic options to help him achieve continued gains on left upper extremity motor function.

Case 2

ER is a 68-year-old right-handed woman with a past medical history of diabetes, high blood pressure, high cholesterol, bilateral internal carotid atherosclerosis, prior stroke 10 years ago affecting her left-side from which she recovered, and recent cognitive and functional decline (co-workers noticed that she was not showing up to work on time as an aide at a nursing home, and not performing tasks at the level that she was before) who was found by her husband, unable to move the right side and unable to speak. She did not present to the hospital in time for acute stroke therapies (tPA or endovascular therapy). She was admitted to the stroke service where her examination showed significant fluctuations in both her right-sided motor strength and language. Stroke work-up showed severe left MCA stenosis and she underwent stent placement, after which her clinical fluctuations improved. MRI showed a shower of ischemic strokes in the left middle cerebral artery territory involving the left frontal and parietal lobes as well as the head of the left caudate and left insula (Figure 1). There was additional evidence of a chronic right parietal infarct, multiple chronic lacunar infarcts, and severe white matter disease.

At the time of acute hospital discharge (ten days post-stroke), she was able to move her right upper extremity against gravity although she had limited coordination and clumsy finger and hand movements. She was able to name simple objects and repeat short phrases but was noted to have limited verbal output. She was transferred to inpatient rehabilitation, where she received three hours of OT, PT, and SLP (one hour each) per day for one month and was noted to have limited clinical improvement over this period. She was subsequently transferred to a skilled nursing facility for two additional weeks and subsequently discharged home. She did receive additional therapy at the nursing facility but the documentation on the frequency and duration were not available (and the patient herself could not recall). She received one month of home therapy (visiting OT, PT, SLP, two times per week), after which therapy was discontinued due to poor participation in activities and lack of clinical improvement.

Six months after her stroke, she was seen in a multi-disciplinary neurorehabilitation clinic (at the same hospital where she received her acute stroke care). She had difficulty arranging an earlier appointment because she lived three hours away and did not have easy access to transportation. She was accompanied by her husband who had to take the day off from work (also worked as an aide at a skilled nursing facility) to bring her to the appointment. Her husband stated that she needed help with nearly all activities of daily living including feeding, dressing, and bathing (he would prepare meals and clothes for her before leaving for work). She was able to feed herself if food was placed in front of her. She used her fingers instead of a fork. She walked for short distances around her home with a walker, independently without supervision, and to get to the bathroom. She spent much of her day seated in front of the TV. The patient denied major mood related issues (i.e., depression, anxiety) but her husband reported low mood observed, and that the patient seemed frequently frustrated.

Examination was notable for very limited expressive language output across all tasks presented. She initiated gestures to support meaning. Auditory comprehension was generally intact for single words and 1-step commands, but there was substantial variability in understanding abstractions or lengthier information presented. There were significant processing speed delays seen throughout the examination. She had a right facial droop and reduced facial movements bilaterally. Response to ideomotor and orobuccal praxis commands were delayed and atypical. In the right upper extremity, tone was normal and she had full active range of motion with near full strength throughout on confrontational testing. She had intact sensation to light touch and proprioception. It was difficult to measure coordination due to her difficulty in initiating movements, and some receptive language impacting her ability to follow motor commands was also noted. She demonstrated perseveration and poor motor planning. In the lower extremities, she had full strength bilaterally. Her gait was characterized by bilateral knee flexion through the stance phase of gait, decreased step length, poor foot clearance through swing phase of gait, and forward truncal lean. She required contact guard to close supervision assist.

The assessment in clinic was that she was substantially more impaired in activities of daily living than her primary impairments in motor and language function would predict. Recommendations were made to attempt to re-initiate home therapy (OT, PT, SLP) with a focus on cognitive-motor interactions. But it was unclear how outpatient therapy or home services could be arranged. Pharmacologic supplementation targeting mood, wakefulness, and motivation was initiated. The plan was to see her back in the neurorehabilitation clinic in six months to assess her progress with these recommendations.
